# Clinical application of genetically modified T cells in cancer therapy

**DOI:** 10.1038/cti.2014.7

**Published:** 2014-05-16

**Authors:** Michael H Kershaw, Jennifer A Westwood, Clare Y Slaney, Phillip K Darcy

**Affiliations:** 1 Sir Peter MacCallum Cancer Centre, Department of Oncology, University of Melbourne, Melbourne, Victoria, Australia; 2 Department of Immunology, Monash University, Prahran, Victoria, Australia

**Keywords:** adoptive cell transfer, chimeric antigen receptor, gene therapy

## Abstract

Immunotherapies are emerging as highly promising approaches for the treatment of cancer. In these approaches, a variety of materials are used to boost immunity against malignant cells. A key component of many of these approaches is functional tumor-specific T cells, but the existence and activity of sufficient T cells in the immune repertoire is not always the case. Recent methods of generating tumor-specific T cells include the genetic modification of patient lymphocytes with receptors to endow them with tumor specificity. These T cells are then expanded *in vitro* followed by infusion of the patient in adoptive cell transfer protocols. Genes used to modify T cells include those encoding T-cell receptors and chimeric antigen receptors. In this review, we provide an introduction to the field of genetic engineering of T cells followed by details of their use against cancer in the clinic.

## Introduction

Lymphocytes of the immune system can eliminate disease with exquisite specificity. This specificity is mediated by antigen receptors expressed by T cells and B cells. The T-cell receptor (TCR) engages antigen presented by major histocompatibility complex molecules on the surface of diseased cells. The B-cell receptor, a form of cell surface-expressed antibody, recognizes native disease-associated molecules expressed on the surface of cells or microorganisms. Although the immune system can eliminate large burdens of infectious disease from the body, processes of immune tolerance and suppression operate in tumors rendering immunity ineffective. Nevertheless, tumor-specific T cells can be isolated from some tumors, and T cells can be activated *ex vivo* to respond against cancer cells.^1^ These T cells can be used effectively as an autologous transfusion in a process termed adoptive immunotherapy.^2^ Melanoma and viral-associated malignancies are particularly responsive to this type of therapy,^3, 4^ and successes in these fields have driven attempts to employ this approach against many types of cancer. Tumor-specific T cells are rare for most malignancies and consequently difficult to isolate, but genetic modification of T cells using genes encoding antigen receptors can be used to generate tumor-reactive T cells in a process termed genetic redirection of specificity.

**Figure 1:**
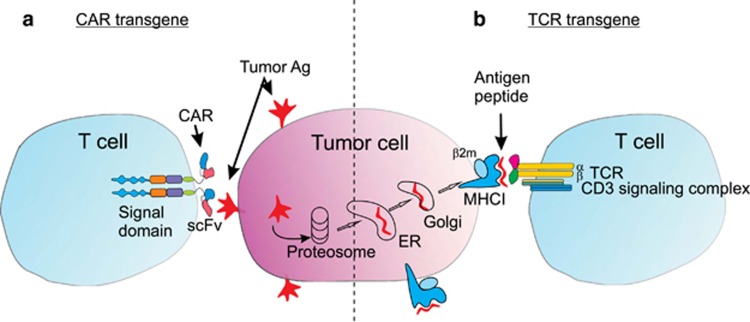
Schematic representation of T cells genetically modified with tumor-reactive CARs or TCR. A tumor cell is shown (center) that expresses an antigen, which can be expressed in its native form on the cell surface or as peptide fragments in the context of major histocompatibility complex I (MHCI) molecules following processing intracellularly by the proteosome, endoplasmic reticulum (ER) and Golgi. (**a**) Cell surface antigen can be recognized by a CAR expressed by T cells. The CAR is composed of an extracellular single-chain antibody domain (scFv) linked by a hinge and transmembrane domains to several intracellular signaling domains, here represented by different colors. CARs are often expressed as dimers, as shown here. (**b**) Intracellularly processed antigen can be recognized by a transgene-encoded TCR expressed by T cells. The TCR associates with endogenous signaling molecules derived from the CD3 signaling complex.

There are two main types of antigen receptors used in genetic redirection (Figure 1). The first utilizes the native alpha and beta chains of a TCR specific for tumor antigen. The second is termed a chimeric antigen receptor (CAR), which is composed of an extracellular domain derived from tumor-specific antibody, linked to an intracellular signaling domain. Genes encoding these receptors are inserted into patients T cells using viral vectors to generate tumor-reactive T cells. This review briefly describes the nature of each type of receptor and its development, followed by a detailed description of the use of TCR and CAR transgenes in the clinic for cancer treatment, in addition to safety considerations and discussion of the future potential of this approach.

## Genetic redirection using TCR genes

There are a number of ways of obtaining genes encoding tumor-reactive TCR. Some antigens are considered relatively immunogenic, and specific TCR can be derived from spontaneously occurring tumor-specific T cells in patients. Antigens included in this category include the melanocyte differentiation antigens MART-1 and gp100, as well as the MAGE antigens and NY-ESO-1, with expression in a broader range of cancers. TCRs specific for viral-associated malignancies can also be isolated relatively easily, as long as viral proteins are expressed by transformed cells. Malignancies in this category include liver and cervical cancer, associated with hepatitis and papilloma viruses, and Epstein-Barr virus-associated malignancies.^5, 6, 7^

Tolerance to most other tumor antigens appears to be too strong to permit isolation of specific TCRs. However, it is possible to obtain TCRs specific for such antigens using several ingenious methods. Allogeneic TCR and transgenic mice expressing human HLA provide an opportunity for the development of tumor-specific T cells away from the tolerogenic environment of the tumor host.^8, 9, 10^ Alternatively, recombinant technology can be used to generate TCRs on phage display libraries, which can be used to identify novel high affinity tumor-specific TCRs.^11^ The antitumor potential of adoptive cell transfer (ACT) using TCR gene-redirected T cells has been demonstrated in mouse tumor models including melanoma, leukemia and prostate cancer.^12^

## Genetic redirection using CAR genes

The specificity of CARs is derived from tumor-specific antibodies, which are relatively simple to generate through immunization of mice. Recombinant techniques can be used to humanize antibodies, or mice expressing human immunoglobulin genes can be used to generate fully human antibodies. Single-chain variable fragments of antibodies are used in the extracellular domain of CARs, which are joined through hinge and transmembrane regions to intracellular signaling domains.

Complete T-cell activation is a complex process involving a primary initiating signal, often referred to as signal 1, and secondary costimulatory signals, often referred to as signal 2. Molecules mediating signal 1 include CD3-ζ that interacts with the TCR, whereas signal 2 molecules include CD28, CD137 and ICOS that interact with ligands on antigen-presenting cells. Together with involvement from coreceptors like CD8 and linker molecules like linker for activation of T cells, triggering of these molecules leads to activation of downstream kinase pathways to promote gene transcription and cellular responses. Although inclusion of primary signaling molecules like CD3-ζ alone in CARs can enable responses against cancer cells, improved responses can be achieved through additional incorporation of signal 2-initiating molecules. Addition of the cytoplasmic domain of CD28, CD134 or CD137 to CD3-ζ-containing CARs can lead to increased cytokine production in response to tumor-associated antigens (TAA) and an enhanced ability of adoptively transferred T cells to mediate tumor regression.^13, 14, 15, 16^

However, being non-major histocompatibility complex restricted in nature, a significant proportion of signals experienced by natural T cells through interaction with antigen-presenting cells are missed using CARs if only signal-initiating molecules alone are triggered. This may lead to deficiencies in some aspects of T-cell biology and sub-optimal responses, as interaction of costimulatory molecules with specific ligands is also necessary for optimal T-cell triggering.^17, 18^

CARs specific for a wide range of antigens have been developed and effective treatment of many different malignancies demonstrated in mice. Cancers targeted in this way include leukemias^19, 20, 21, 22^ and solid cancers including cancers of the breast,^23^ pancreas,^24, 25^ ovaries^26^ and others.^27^

The TCR and CAR approaches each have advantages and limitations compared with the other, which need to be considered in the context of individual disease settings. For example, TCR can detect both intracellular and cell surface TAA, and can harness the entire signaling network normally engaged by TCR. In addition, TCR can enable activation, costimulation and expansion of T cells through interaction with antigen-presenting cells. However, TCR are restricted by major histocompatibility complex and so each TCR is applicable to only a proportion of patients, and transgene TCR can be mispaired with endogenous TCR reducing their specificity and activity.^28^

A CAR on the other hand, responding in a non-major histocompatibility complex-restricted manner, can potentially be used for all patients, but they can generally only detect cell surface TAAs, which can include carbohydrate moieties and glycolipids, major classes of molecules and potential sources of TAA.^26^ In addition, only a proportion of normally recruited signaling components are used, and engagement of antigen-presenting cells is not effective. However, the cassette-like nature of CAR structure enables the inclusion of additional signaling moieties to somewhat address these issues.

## Clinical application of gene-redirected T cells

Since the initial conception of the idea to genetically redirect T cells in 1989,^29^ a large amount of work has been performed *in vitro* and in mouse models using ACT against cancer.^30^ The extraordinary promise of ACT derived from these studies has led to clinical application, with the first reported trial of work beginning in 1996 in patients with ovarian cancer.^31^ More recently, a wide variety of cancers have been targeted using gene-modified T cells and some remarkable responses have been reported using either genes encoding TCRs or CARs (Table 1).^12^

## Clinical trials using TCR gene-modified T cells

The first opportunity to test TCR-redirected T cells was afforded by the isolation of an anti-MART-1-specific TCR from lymphocytes infiltrating melanoma.^32, 33, 34^ Objective responses were observed in 13% of 31 patients treated with gene-modified T cells. These studies provided encouragement for further development of this approach. However, the response rate was lower than that observed in clinical trials using naturally occurring tumor-infiltrating lymphocytes (TIL) of diverse specificities.^2^

In an attempt to increase the efficacy of treatment, higher avidity TCRs were developed with specificities for the melanoma-associated antigens MART-1 and gp100. Objective tumor responses were reported in 33% of 20 patients and 13% of 16 patients using T cells redirected against MART-1 and gp100, respectively.^35^ Interestingly, toxicity against normal tissues expressing the antigens was observed, which included cells in the skin, ear and eye. Toxicity was managed using corticosteroid treatment in these studies.

Extension of TCR gene-modified T cells to other cancers was afforded by the isolation of a Carcinoembryonic antigen (CEA) specific TCR gene from HLA transgenic mice.^8^ A partial response was observed in one of three patients treated, and decreases in levels of circulating CEA observed in all patients. However, major toxicity was observed against normal gut epithelium expressing CEA leading to severe colitis in all patients. Colitis was transient, but this study was ceased due to toxicity concerns. These studies highlighted the need to choose antigen targets with little, if any, expression on vital normal tissues.

Cancer–testis antigens can have very limited expression on normal tissues and as such represent a reasonable target antigen. NY-ESO-1 is a cancer–testis antigen and a TCR encoding activity against this antigen was used to redirect T cells against tumors in patients with melanoma or synovial cell carcinoma.^36^ Partial responses were observed in 66% of sarcoma patients and 27% of melanoma patients. This study confirmed NY-ESO-I as a safe and effective target antigen for T-cell therapy, and larger trials targeting this antigen are justified.

Nevertheless, not all cancer–testis antigens are safe targets, as demonstrated in two recent clinical trials targeting the MAGE-A family of antigens. In a trial using a TCR specific for MAGE-A3/A9/A12, five of nine patients achieved tumor regression.^37^ However, three of nine patients experienced neurologic toxicity that led to death of two patients. Subsequent analyses identified previously unknown expression of MAGE-A12 in human brain tissue as the likely cause of toxicity. In another phase I trial using another TCR targeting MAGE-A3, two patients experienced cardiovascular toxicity after receiving TCR gene-modified T cells for the treatment of melanoma or myeloma, leading to death.^38, 39^ Subsequent investigations revealed a previously undefined cross-reactivity of the MAGE-A3 TCR with a muscle-specific protein, Titin, expressed in the heart. These studies indicate a cautious approach to the use of genetically redirected T cells and highlight the need for robust assessment of safety before clinical trial.

## Clinical trials using CAR gene-modified T cells

The first clinical trials using ACT of CAR-modified T cells were performed in patients with ovarian cancer, colon cancer, neuroblastoma and renal cancer^31, 40, 41, 42, 43^ (Table 1). Despite the transfer of large numbers of T cells in some cases, there were no significant responses reported, except for one partial response in a patient with neuroblastoma.^42^ CARs employed in these earlier trials had signaling domains derived from a single molecule, CD3-ζ or FcR-γ, and are referred to as first-generation CARs. The poor response rate may have been due to sub-optimal triggering of T-cell activity by the first-generation CARs, and further studies using second- or third-generation CARs containing signaling domains from multiple molecules may produce better responses. Another potential reason for the lack of responses in these first trials may be due to the use of murine single-chain antibodies in CAR design, which resulted in CAR neutralization by human anti-mouse antibody formation in patients.^31, 44^

In general, it has been difficult to demonstrate CAR T-cell activity in the clinic against solid cancer (Table 1), although some recent studies in neuroblastoma led to objective tumor responses in 3 of 19 patients.^45^ Prostate cancer may also be responsive to CAR T cells with a report of two partial responses from five patients in a clinical study using CAR T cells redirected toward PSMA.^46^ Persistence of adoptively transferred T cells can be low in these studies, often as short as several days or weeks,^31, 42^ and patient preconditioning with lymphodepletion using cyclophosphamide and fludarabine may enhance engraftment of transferred cells.^2^

Some toxicity has also been observed following CAR T-cell transfer for cancer treatment if the target antigen is expressed on normal tissues. For example, toxicity was observed against liver following transfer of T cells bearing a CAR specific for carbonic anhydrase (CA) IX on renal cell carcinoma, due to expression of the antigen by bile duct epithelium.^47^ However, toxicity was reduced using a strategy involving administration of anti-CAIX before transfer of gene-modified T cells.^40^

More impressive clinical responses have been observed using CAR T cells against blood cancers, with significant reduction in disease reported for various lymphomas and leukemias of B-cell origin (Table 1). Response rates as high as 60–85% have been achieved targeting CD19 expressed on a variety of B-cell leukemias and lymphomas.^48, 49, 50, 51, 52^ Expansion of transferred CAR T cells can exceed 100 000-fold to be over 50% of circulating lymphocytes,^49, 53^ with complete disease responses associated with expansion to over 5% of circulating CD3^+^ lymphocytes.^54^ The ability of transferred T cells to persist long term, over 3 years, has also been demonstrated.^54^

Donor-derived T cells can also be used for genetic modification with CARs following allogeneic stem cell transplantation.^49, 51^ Although there may be an increased risk of GVHD after donor-derived T-cell transfer, this risk can be reduced by using CAR-expressing viral-specific T cells, which have the added benefit of antiviral activity.^55^

Of concern in targeting CD19 is expression of this molecule on normal B cells. Indeed, normal B cells can be destroyed by transferred T cells and B-cell aplasia has been observed in several studies. Patients can be predisposed to infection from the resulting hypogammaglobulinemia, although this can be managed with intravenous immunoglobulin.^49^ More selective approaches for elimination of tumor cells include targeting the kappa light chain of surface immunoglobulin expressed by many hematologic malignancies.^56^ This approach may provide better specificity that preserves normal B cells expressing lambda light chains.

Acute toxicities can occur following T-cell transfer, which include cytokine release syndrome, resulting from T-cell activity against antigen.^48,52^ Toxicity can be concerning, but can be managed using steroids or an antibody neutralizing IL-6.^48, 49^ The severity of cytokine release syndrome varies between patients, but the level of serum cytokines have not correlated with patient response to date.^57^ Adverse events can also include tumor lysis syndrome, associated with rapid reduction of large tumor burdens.^51^ Uric acid release during tumor lysis contributes to kidney toxicity, which can be managed with rasburicase.

Trafficking of adoptively transferred CAR T cells has been demonstrated to sites of disease including skin and bone marrow,^58^ with some preliminary evidence that disease in the marrow can be more easily eliminated than disease in lymph nodes.^59^

## Safety measures using genetically redirected T cells

Despite the promising results emerging from early phase clinical trials, several groups have reported on-target toxicity to normal tissue following adoptive transfer of CAR or TCR gene-modified T cells in patients.^8, 60^ The recent deaths of patients treated with either anti-erbB2 CAR T cells^61^ or T cells engineered to express a TCR specific for MAGE-A3^37, 39^ highlight the need to develop new safeguard measures for this type of therapy. A number of different strategies have been trialed to selectively eliminate genetically modified T cells. This includes the introduction of a conditional ‘suicide' gene such as the herpes simplex thymidine kinase (*HSV-tk*) gene. Expression of *HSV-tk* is able to convert nucleoside analogs, such as the antiviral drug ganciclovir, into products that cause death of the dividing cell. Notably, however, it was reported in a clinical trial that not all transferred T cells expressing the *HSV-tk* gene were effectively eliminated.^62^ In other studies, the *HSV-tk* gene was found to be immunogenic leading to rapid deletion of adoptively transferred T cells in patients,^63^ and silencing of the *HSV-tk* gene can lead to emergence of ganciclovir-resistant T cells in mice.^64^

Given these problems, other novel suicide genes have been designed. The development of apoptosis-inducing fusion proteins such as inducible Fas and Caspase 9, which can be activated through the use of chemical inducers of dimerization, shows promise.^65^ Expression of the gene in T cells allows for the selective elimination of gene-modified T cells. Other approaches for elimination of gene-modified T cells involve expression of CD20 or truncated epidermal growth factor receptor in T cells. Administration of cell-depleting antibodies, rituximab or cetuximab, can lead to elimination of human T cells engineered with these molecules.^66, 67^

However, a potential constraint with all of these approaches is the need to be able to effectively express both the antigen receptor transgene and suicide gene at high levels, which is a challenge for current gene transfer technologies. An innovative approach to circumvent this problem, by incorporating the selectable gene within the antigen receptor, has involved transducing T cells with myc-tagged TCRs.^68^ The use of a tag-specific antibody effectively depleted the transduced T cells in mice and unlike other strategies is not affected by low expression of the transgene. The validity of these various strategies will need proper evaluation in future clinical trials.

A potential problem for T cells modified with new TCRs is for the transgene to mispair with endogenous TCRs and form hybrid receptors with unknown specificity causing autoreactivity.^69^ Importantly, this has not been observed in the clinic to date.^70^ Nevertheless, it is still desirable to reduce mispairing to increase expression of tumor-specific TCR as described above, which should also eliminate any concerns of potential autoreactivity.

Another safety concern using some retroviruses to genetically modify T cells is their propensity to integrate near start sites of genes, which could lead to gene dysregulation, cell transformation and oncogenesis, as observed in gene-modified stem cell transfer.^71^ However, there have been no transformation events reported to date in mice or patients following retroviral transduction of T cells.^72, 73^ Nevertheless, alternative gene transduction methods with less tendency to insert near active genes have been utilized with success in both mouse models and patients, including the use of lentiviral vectors,^74^ nonviral transposon systems^75, 76^ and direct RNA electroporation.^77^ An additional safety feature of using RNA transfection is the short expression period of several days, meaning that any transgene-mediated toxicity abates after expression is lost. Along similar lines, expression of transgenes could be more tightly controlled by using inducible promoters to drive antigen receptor expression.^78^

T-cell activity against normal tissues can also be controlled by expressing more than one CAR in T cells. For example, targeting two TAA simultaneously can deliver higher responses against tumor cells, as long as normal cells express only one of the antigens.^79^ Combinatorial signaling between two CARs can also be used to more accurately direct T cells against tumors. In this approach, signal 1 and signal 2 are delivered through separate CARs specific for two TAA, and maximal responses are elicited only when two TAA are present.^80, 81^ Using these kind of approaches, bar codes can effectively be assigned to tumor and normal tissue cells and T-cell responses delivered only when they ‘read' the correct bar code.

## Concluding remarks

Cell selection also affords an opportunity to enhance the anticancer potential of ACT. Although T cells have been the main focus of efforts to provide tumor-reactive cells, largely due to their ease of manipulation, there is evidence that other types of genetically redirected cells can mediate tumor inhibition.^82^ For example, the genetic modification of other types of lymphocytes such as NK cells and gamma/delta T cells can yield cells able to respond against tumor cells.^83, 84^

The immunosuppressive nature of the tumor microenvironment remains a significant hurdle to T-cell therapies. Further approaches aimed at genetically neutralizing specific immunoregulatory mechanisms or the addition of reagents targeting immune checkpoints to adoptive cell therapies may overcome immune suppression and enhance T-cell-mediated tumor inhibition.^23, 85, 86^

Following exposure of T cells to antigen, differentiation is thought to proceed from a naive state through stem cell memory, central memory and effector memory phenotypes before differentiation to effector T cells. The use of less-differentiated T cells, in particular stem cell memory T cells, represents another option for enhancing the survival of tumor-specific T cells.^87^ In addition to the phenotype of transferred T cells, the manipulation of their mode of delivery may lead to enhanced persistence, at least for some malignancies, as demonstrated in a recent novel approach using fibrin matrices.^88^

Given the critical requirements for trafficking, future strategies to enhance this aspect of tumor immunity will be important for optimal T-cell responses against tumors. Although most efforts at genetically redirecting migration of T cells have focused on chemokine receptors, future additional modification with genes encoding integrins or their ligands may further enhance tumor-specific homing.^89^ Improvements in T-cell penetration of tumors can also be afforded by blocking inhibitors of migration such as endothelin^90^ or by using irradiation to normalize the often chaotic structure of tumor blood vessels.^91^ Combining these latter strategies with adoptive transfer of genetically redirected T cells may increase their localization to tumors where they can mediate destruction of tumor cells.

In conclusion, abilities of T cells to specifically lyse tumor cells and secrete cytokines to recruit and support immunity against cancer make them an attractive proposition for therapy. The ability to genetically modify T cells to respond specifically against tumors has broadened the range of malignancies for which this therapy could be used. Recent descriptions of complete responses of hematologic malignancies to adoptive transfer of T cells is generating much excitement and provides optimism for the use of genetically engineered T cells for treatment of common solid cancers.

## Figures and Tables

**Table 1:** Published reports of clinical trials using genetically redirected T cells for cancer therapy

*Cancers*	*Target antigens*	*Antigen receptor*	*Year reported*	*Number of patients*	*Responses*	*References*
AML	Lewis Y	CAR	2013	4	0	^58^
Colorectal and breast	CEA	CAR	2002	7	Minor response in two patients	^41^
Colorectal	CEA	TCR	2011	3	1 PR	^8^
	Her-2	CAR	2010	1	0	^61^
	TAG-72	CAR	1998	16	1 SD	^43^
Leukemia and lymphoma	CD19	CAR	2013	10	1 CR, 1 PR, 1 SD	^51^
	CD19	CAR	2013	5	1 SD	^76^
	CD19	CAR	2013	20	14 CR	^49^
	CD19	CAR	2013	13	10 CR	^48^
	CD19	CAR	2013	8	5 CR	^52^
	CD19	CAR	2013	6	2 CR, 2 SD	^59^
	CD19	CAR	2013	24	5 CR, 7 PR	^54^
	CD19	CAR	2013	20	6 CR, 11 PR, 1 SD	^53, 92, 93^
	CD19 and CD20	CAR	2010	4	0	^94^
	CD19	CAR	2011	6	2 SD to 10 months	^95^
	CD20	CAR	2008	7	1 PR, 4 SD, 2 NED maintained	^96^
	CD20	CAR	2012	3	1 PR, 2 NED maintained	^96^
Melanoma	gp100	TCR	2009	16	1 CR, 2 PR	^35^
	gp100	TCR	2010	10	NI	^70^
	MART-1	TCR	2006	15	1 PR	^33^
	MART-1	TCR	2009	31	4 OR	^32, 37^
	MART-1	TCR	2009	20	6 PR	^35^
	p53	TCR	2010	14	NI	^97^
Melanoma, esophageal and synovial sarcoma	MAGE-A3	TCR	2013	9	1 CR, 4 PR	^37^
Melanoma and sarcoma	NY-ESO-1	TCR	2011	17	2 CR, 7PR	^36^
Multiple myeloma	NY-ESO-1	TCR	2012	11	3 CR, 7 PR	^98^
Neuroblastoma	CD171	CAR	2007	6	1 PR	^42^
	GD2		2011	19	3 CR	^45^
Ovarian	αFR	CAR	2006	12	0	^31^
RCC	CAIX	CAR	2011	11	0	^40, 44^
Prostate	PSMA	CAR	2013	5	2 PR	^46^

Abbreviations: CR, complete response; NED, no evidence of disease; NI, no information; OR, objective response; PR, partial response; RCC, renal cell carcinoma; SD, stable disease.
